# The Woman Workers' World

**Published:** 1918-07-27

**Authors:** 


### THE WOMAN WORKER'S WORLD.

The idea that "continued education" means forcing a number of recalcitrant young people back to the schoolroom to "do lessons" after the day's work, can best be corrected by tangible results. A large party of parents and friends recently gathered at Exmouth Street Centre to see the exhibition and demonstration of afternoon work by Messrs. Crosse and Blackwell's girl employees. After some excellent breathing and voice-producing exercises, Handel's "Where'er you walk" was charmingly sung, the appreciation of "good" music being evident. The dancing and physical exercises were also excellent; the onlooker would not be surprised to learn that the best testimony is given by increased weight and measurement.

The cookery exhibit included good soup, stews, some attractive oatmeal pastry filled with cherries, curried eggs, and other war-time and other-time dishes. The needlework also showed the soundness of the instruction, which is given by L.C.C. teachers, construction and choice of material being taught, as well as simplicity and suitability of decoration. Comfortable and pretty garments for the active worker were fashioned in serge, holland, etc., as well as some holiday wear and children's garments, the ornamentation consisting chiefly of a little embroidery in good design on the material, generally in colours, and borders of handwork.

The chair was taken by Mr. Frank Blackwell, and some book-prizes presented. If the example set by this firm can be widely copied, with or without an Education Act, great good must result. Such action is patriotic, involving expense, for eight hours weekly are compulsory for employees of fourteen to sixteen, and till eighteen one weekly lesson of two and a half hours is now optional. Tea is provided by the firm, and study lasts from five to seven on the first four week-days. Two hours are given to needlework or dressmaking, two to laundry for two terms, cookery four terms, one hour drill, one and a half hour music, half hour arithmetic, and one hour "English."

Cucumbers are now plentiful, but refused by many people as unwholesome. But if cut in half, or, if very long, in three pieces, which are then stuffed and served hot, they are an excellent war-time dish. Scoop out the seeds from each piece, leaving the bottom fairly thick, then plunge in boiling water for five minutes and drain. Stand in casserole or greased baking-dish, make a stuffing with tomato, chopped onion, breadcrumb, a little lemon-juice, butter, or fat. Cover with crumbs and grated cheese; bake till brown, basting a little while cooking.

